# Unravelling the impacts of captivity on saltwater crocodile (*Crocodylus porosus*) cloacal bacterial communities and physiology

**DOI:** 10.1093/femsec/fiaf114

**Published:** 2025-11-19

**Authors:** T Franciscus Scheelings, Saritha Kodikara, David J Beale, Thi Thu Hao Van, Robert J Moore, Lee F Skerratt

**Affiliations:** Melbourne Veterinary School, Faculty of Science, The University of Melbourne, Werribee, Victoria 3030, Australia; School of Mathematics and Statistics, Faculty of Science, The University of Melbourne, Parkville 3052, Australia; CSIRO, Ecosciences Precinct, Dutton Park, Queensland 4102, Australia; School of Science, RMIT University, Bundoora West Campus, Bundoora, Victoria 3083, Australia; School of Science, RMIT University, Bundoora West Campus, Bundoora, Victoria 3083, Australia; Melbourne Veterinary School, Faculty of Science, The University of Melbourne, Werribee, Victoria 3030, Australia

**Keywords:** crocodile, gut, metabolome, microbiota, physiology

## Abstract

This study addresses a significant research gap in understanding the impacts of captivity on the bacteriome and physiology of saltwater crocodiles (*Crocodylus porosus*). Despite their ecological and cultural significance, crocodilians are a taxon that remains underexplored in microbiome research. We investigated cloacal bacteriome samples from both wild and captive populations to identify compositional and functional differences resulting from captivity. Our findings reveal significant alterations in bacterial diversity and community structure in captive crocodiles, with notable shifts at both phylum and family levels; specifically, Bacteroidota and Fusobacteriota dominate in captivity, whereas wild crocodiles exhibit a higher prevalence of Pseudomonadota and Bacillota. The Shannon diversity index indicates a significant reduction in bacterial diversity among captive individuals, likely due to husbandry practices that foster a microbially depauperate environment. Additionally, serum metabolomics analysis shows an enrichment of alcohol sugars in captive crocodiles, alongside a decrease in pantothenic acid. While this is the first study to characterize these traits in saltwater crocodiles, further research is necessary to determine the physiological consequences of these bacterial and metabolic changes on host fitness and adaptability. Longitudinal studies are essential for understanding how bacterial communities evolve over time and in response to environmental factors, which will inform conservation strategies and improve the management of captive populations of crocodilians intended for reintroduction into the wild.

## Teaser

Examination of the cloacal bacterial communities between captive and wild crocodiles revealed significant differences.

## Introduction

The gastrointestinal tract of vertebrates harbours an intricate community of microorganisms known as the microbiota. The inhabitation of metazoans by these symbionts that has played an essential role in the expansion of Earth’s biodiversity (Lee and Mazmanian 2010, McFall-Ngai et al. 2013). Hosts and their microbiotas form a mutually beneficial holobiont, with their distinct combined genetic signatures termed the hologenome (Theis et al. 2016). This multigenomic ecosystem is now considered essential for animal homeostasis, blurring the lines between the individual and its microbial residents. Consequently, animals can be viewed as highly complex biological ‘super organisms’, where a significant portion of physiological function arises from microbial activity (Dethlefsen et al. 2007).

The gut microbiota of vertebrates is malleable, and composition is ultimately governed by a complex interplay of intrinsic and extrinsic characteristics including genetics (Zhao et al. 2013), diet (Nelson et al. 2013, Frese et al. 2015, Henderson et al. 2015), habitat quality (Amato et al. 2013), geography (Scheelings et al. 2020a), sex (Stoffel et al. 2020), ontogeny (Price et al. 2017), and phylogeny (Scheelings et al. 2020b). A further confounding factor is that captivity has been shown to play a significant role in shaping animal microbial communities in all vertebrate classes (McKenzie et al. 2017, Florkowski et al. 2023, Sun et al. 2024, Tong et al. 2019, Tang et al. 2020), which may have negative implications for captive breed and release programs for conservation efforts (Dallas and Warne 2023). This is particularly pertinent for crocodilians, where 50% of the 28 extant species are listed as Threatened (Griffith et al. 2022, Lourenco-de-Moraes et al. 2023), and for which some species such as the Philippine crocodile (*Crocodylus miondorensis*), Orinoco crocodile (*Crocodylus intermedius*), African dwarf crocodiles (*Osteolaemus* spp.), gharial (*Gavialis gangeticus*), and the Siamese crocodile (*Crocodylus siamensis*), *ex situ* programs form an integral component of their conservation (Manalo and Alcala 2014, Schmidt et al. 2014, Chattopadhyay et al. 2019, Khadka et al. 2022, Saldarriaga-Gómez et al. 2023). Thus, it is important to understand how altered microbiotas may affect crocodilian physiology in animals that have been intended for reintroduction into the wild.

Despite their cultural and ecological significance, crocodilians are one of the most poorly studied taxa for microbiota investigations. A likely limitation is that crocodilians can be large, powerful animals, and in the case of saltwater crocodiles (*Crocodylus porosus*) are dangerously aggressive towards humans (Manolis and Webb 2013), which poses logistical challenges when working with them. Studies have been predominately limited to captive specimens of saltwater crocodiles (Willson et al. 2019, Siddiqui et al. 2023, Beale et al. 2025), American alligators (*Alligator mississippiensis*) (Perez-Marron et al. 2022), and Siamese crocodiles (Lin et al. 2019), with the only investigations involving wild animals occurring in Chinese alligators (*Alligator sinensis*) (Hu et al. 2022, Tang et al. 2019), and the American alligator (Keenan et al. 2013). Additionally, most of these studies have been descriptive in nature, which makes inference on the physiological effects of the host–microbiota relationship difficult. While identifying the specific microbes and their varying abundances in hosts is crucial, it is also fundamentally important to appreciate their impact on host physiology. One way of doing this is by using a mutiomics approach, where microbial functional analyses are incorporated into the dataset (Nyholm et al. 2020).

Comprehending crocodilian microbiomes is necessary in the context of vertebrate evolution as these ancient reptiles serve as a key link in the evolutionary chain of archosaur divergence (Nesbitt et al. 2013). Their unique microbiotas, shaped by their inimitable natural history, offers insights into the symbiotic relationships that have developed between host and microbe over eons. By studying the microbial communities within crocodilians, we can uncover how these organisms contribute to health and homeostasis, which may reflect adaptations seen in early vertebrates. Additionally, examining crocodilian microbiotas can shed light on the ecological roles they play in their environments, potentially revealing evolutionary strategies that have persisted or transformed through time. This understanding not only enhances our knowledge of crocodilian biology but also provides a broader perspective on vertebrate evolution.

The goals of this investigation were to examine the cloacal bacterial communities of both captive and wild saltwater crocodiles and to assess their functional impact on physiology. By employing a multiomic approach, we aim to deepen our understanding of the bacteriome’s role in this understudied taxon and to shed light on the coevolutionary history between vertebrates and their microbiota.

## Methods

### Study populations

This study was approved by The University of Melbourne Office of Research Ethics and Integrity (Ethics ID: 2022–24808–32226–4) and all experiments were performed in accordance with relevant guidelines and regulations. Crocodiles were trapped and sampled under the permit 72 249 from the Parks and Wildlife Commission of the Northern Territory, Australia.

Adult, free-ranging saltwater crocodiles (*n* = 8) were trapped in various locations within the Darwin Harbour area, as part of the Northern Territory’s Problem Crocodile Management Program (Fukuda et al. 2014) in April 2023. Crocodiles were captured in marine waters using large aluminium traps suspended in the water with flotation devices and baited with sections of wild pig. Traps were checked three times per week, depending on local tide and ocean conditions. The exact duration each crocodile spent in the trap was unknown, with some individuals potentially confined for up to 3 days. Limited trapping success during the sampling period resulted in lower-than-anticipated capture rates. Due to the significant logistical challenges, costs, and safety risks involved in working with wild saltwater crocodiles, it was not feasible to extend sampling beyond the initial field effort. When a crocodile was found within a trap, it was removed using ropes attached to the jaws and then restrained by taping the mouth shut and covering the eyes with gaffer tape. Mobility was further restricted by securing the hind limbs with ropes. The crocodile was then transported to a central processing hub for sampling where travel time could take anywhere from 30 to 90 min depending on the location of the trap, and ocean and wind conditions. Following sample collection, the crocodile was collected by a private enterprise for commercial use. No attempt was made to assess the diet of wild crocodiles but it has been shown that animals in the Northern Territory have a heavy reliance on terrestrial prey such as water buffalo (*Bubalus bubalis*) and pigs (*Sus scrofa*) (Adame et al. 2018). However, animals moving in and out of Darwin Harbour are probably not actively feeding but would be attracted to carrion if it is available (Webb, personal communication).

Adult captive crocodiles (*n* = 20) were all sampled at a privately owned zoological institution in Darwin. Crocodiles were housed in a large freshwater enclosure in which ~50 animals resided. The night before capture the central pool was drained over the course of ~8–10 h to enable hand capture of animals. Individual crocodiles within this enclosure were randomly selected for sampling and were manually restrained using ropes and tape in a similar manner to the wild animals. Time from capture to sampling was ~15 min, and it took roughly 7 h to sample all 20 crocodiles. After sampling, all captive crocodiles were permitted to return to the enclosure and the pool was refilled with water. Captive crocodiles were fed mostly chicken heads and necks as young animals, which progressed to include larger crab, fish, pig, and chicken carcasses as the animals increased in size and age.

### Sample collection

Following restraint, an equine uterine swab (Minitube) was inserted into the cloaca. These swabs are ~90 cm in length, and each swab was inserted to a depth of at least 40 cm. These swabs were housed in a sterile sheath, the entire apparatus was inserted into the cloaca, and the swab tip was extruded when correct placement of the sheath had been achieved. The swab tip was then retracted back into the sheath prior to extraction to ensure that it had no contact with the external environment and removed from the cloaca. The tip of the swab was then cut using flame-sterilized wire cutters and stored in 1 ml of ZymoBIOMICS DNA/RNA Shield (Integrated Sciences) in a sterile Eppendorf tube. The Eppendorf tubes were then frozen and stored at −80°C until DNA extraction could occur.

Once cloacal swabbing had been completed, 10 ml of blood was collected from the ventral coccygeal vein via a lateral approach using a 10 ml syringe attached to an 18-gauge needle. Immediately after collection, blood was transferred into a gel clot activator tube (Sarstedt AG & Co., Nümbrecht, Germany). Blood tubes were then placed into a portable ice pack and taken back to the laboratory for processing. Once back at the laboratory, the tube was centrifuged, and the resultant serum removed and stored at −80°C until analysis

### Microbial DNA extraction

DNA was extracted using the ZymoBIOMICS DNA Miniprep Kit (Integrated Sciences) according to the manufacturer’s instructions. Following extraction, DNA was stored at −80°C until amplicon sequencing could take place. Blank swabs were used as negative controls and underwent DNA extraction the same as sample swabs.

### 16S rRNA gene amplicon sequencing

The V3–V4 region of 16S rRNA genes were Polymerase Chain Reation (PCR) amplified with forward primer 5′ ACTCCTACGGGAGGCAGCAG 3′ and reverse primer 5′ GGACTACHVGGGTWTCTAAT 3′ using Q5 high fidelity polymerase (New England Biolabs) with a dual barcoding strategy (Fadrosh et al. 2014). The PCR cycling parameters were, 98°C for 1 min, 35 cycles of 98°C for 10 s, 49°C for 30 s, and 72°C for 30 s, followed by a 10-min extension at 72°C. Sequencing was performed on an Illumina MiSeq system (2 × 300 bp).

### Data processing

Sequence data was analysed using Quantitative Insights into Microbial Ecology 2 (QIIME2) version 220.6 (Bolyen et al. 2019), using the Divisive Amplicon Denoising Algorithm (DADA2) plugin for quality filtering, denoising, chimaera detection, and amplicon sequence variant (ASV) calling (Callahan et al. 2016). ASVs were taxonomically classified using the SILVA database (v138.1) (Quast et al. 2013). An ASV abundance table with taxonomic assignments was produced for further analysis.

### Functional omics analysis

Metabolites and lipids were extracted from 100 μl of crocodile serum using a one-pot extraction method previously described (Beale et al. 2022). Briefly, serum was quenched with 450 μl of ice-cold (−20°C) methanol: ethanol (50 % v/v; LiChrosolv®, Merck, Darmstadt, Germany), and vortexed for 2 min. The samples were centrifuged (Centrifuge 5430R, Eppendorf, Hamburg, Germany) at 14 000 rcf at 4°C for 5 min to pellet the protein. The supernatant was transferred and filtered using a positive pressure manifold (Agilent PPM48 Processor, Agilent Technologies, Santa Clara, California, USA) with Captiva EMR cartridges (40 mg, 1 ml; Agilent Technologies, Mulgrave, VIC, Australia) to separate the lipid and metabolite fraction (Beale et al. 2022). Two stable-isotope internal standards were used throughout the extraction; 100 ppb of l-Phenylalanine (1–^13^C) was spiked first with the EtOH: MeOH, followed by a 200 ppb of Succinic Acid (1,4–^13^C_2_) added when samples were reconstituted. The internal standards were sourced from Cambridge Isotope Laboratories (Andover, MA, USA). The residual relative standard deviation (RDS%) of the raw internal standards were 0.5 % (l-Phenylalanine, 1–^13^C) and 0.6 % (Succinic Acid, 1,4–^13^C_2_).

Central carbon metabolism metabolites were analysed on an Agilent 6470 LC-QqQ-MS coupled with an Agilent Infinity II Flex UHPLC system (Agilent Technologies) using the Agilent Metabolomics dMRM Database Method (Gyawali et al. 2021). Untargeted nonpolar lipids were analysed using an Agilent 6546 Liquid Chromatography Time-of-Flight Mass Spectrometer (LC-QToF) with an Agilent Jet Stream source coupled to an Agilent Infinity II UHPLC system (Agilent Technologies) (Beale et al. 2021 efer, Gyawali et al. 2021, Shah et al. 2022). Lipid annotation was performed using acquired Auto MSMS data on pooled PBQC samples obtained at 20 eV and 35 eV collision energy. Collected Auto MSMS data were then processed using the MassHunter Lipid Annotator tool (version 1.0, Agilent Technologies), and a sample specific PCDL database was created and used to putatively identify lipids in all analysed samples based on MSMS spectra and library threshold score of 0.8 (Demichev et al. 2020).

### Statistical analyses

ASV abundance data was analysed in R, utilizing the package ‘phyloseq’ (version 1.50.0) (McMurdie and Holmes 2013). Alpha diversity was explored using Observed ASVs, Inverse Simpson and Shannon index estimates. Alpha diversity was tested for normality using the Shapiro–Wilks test and when data was nonnormally distributed comparisons between locations were made using the Wilcoxon rank-sum test, and for normally distributed data comparisons between sites were made using the Welch two sample *t*-test. To account for differences in numbers between groups we then calculated effect sizes for all alpha diversity metrics, where *r* = 0.1 (small effect), *r* = 0.3 (medium effect), and *r* = 0.5 (large effect). Beta diversity was investigated at the family level using principal coordinate analysis (PCoA) on Bray–Curtis distances and we used the adonis2 function from the R package ‘vegan’ (version 2.6–10) to perform PERMANOVAs to compare beta diversity between captive and wild crocodiles, and we tested for homogeneity of dispersion using beta dispersion (Oksanen et al. 2025). For abundance testing read counts were transformed to proportions per sample [i.e. total sum normalization (TSN)] prior to calculating the distances and dissimilarities (McKnight et al. 2018).

To investigate the relationships between microbiota composition and metabolomic profiles, we performed a multiomics analysis across the entire dataset using the R package ‘mixOmics’ (version 6.30.0) (Rohart et al. 2017). For this analysis, we divided our omics data into metabolomics (amino acids, nucelotides, organic acids, alcohols, vitamins, and carbohydrates) and lipidomics. Due to the long chemical structure names assigned to lipids we renamed these compounds using a simple numbering system, the original names can be found in the supplementary material (Table S1). First, performed cumulative sum scaling normalization on the microbiota data to correct for any bias in the assessment of differential abundance that may have been introduced by TSN (Rohart et al. 2017). We then filtered all data (microbiota, metabolomics, and lipidomics) by removing low counts of <0.01% and we chose to assess microbiota data at a Family level due to high sparsity at ASV level. We used DIABLO (Data Integration Analysis for Biomarker discovery using Latent-variable approaches for Omics studies) (Singh et al. 2019), to integrate multiomics data from our 28 crocodiles, comparing captive versus wild animals. This linear, supervised method is well-suited for small cohorts and binary traits, as it limits model complexity and reduces overfitting. DIABLO maximizes shared signal across omics layers while selecting a sparse, discriminative feature set. Its built-in cross-validation tunes model components and sparsity penalties for reproducible results (Rohart et al. 2017). Recent benchmarks recommend DIABLO for small-sample studies, with Sparse Integrative Discriminant Analysis (Safo et al. 2022) preferred for larger datasets (Novoloaca et al. 2024). Unsupervised methods [e.g. MultiOmics Factor Analysis (Argelaguet et al. 2018), Similarity Network Fusion (Wang et al. 2014), autoencoders (Miotto et al. 2016)] are better suited for uncovering hidden substructure (Rönn et al. 2024). Given our small sample size and clear group contrast, DIABLO is a robust choice for supervised, exploratory multiomics integration. We explored correlations within and between selected features across datasets and examined these associations in the first two components. To visualize the relationships among selected features, we generated a circos plot, where different feature types are arranged in a circular layout, with links representing strong positive or negative correlations (*r* > 0.7). Additionally, we created a clustered image map (CIM) to further explore the multiomics molecular signature expression across samples. Finally, we evaluated model performance using five-fold cross-validation repeated 10 times to identify the optimal number of components (Fig. S1). Five folds were selected to ensure balanced representation of both groups within each fold, given the smaller number of Wild individuals (*n* = 7). Classification error rates were weighted based on the correlation between predicted co as well as weighting the classification error rate from each dataset according to the correlation between the predicted components and the Y outcome (Fig. S2). For all statistical analyses random seeds were set at 999 and significance was accepted if *P* < .05.

To determine the origin of metabolites (host, microbe, environment, or cometabolism) we used the online resource MetOrigin (Yu et al. 2022). We did this for all explanatory metabolites for components 1 and 2 as predicted by our model. MetOrigin has a reference dataset for the saltwater crocodile as the host species, however not all our metabolites were able to be assigned a KEGG or Human Metabolome Database identification and therefore these have been excluded from this analysis.

## Results

### Microbiota results

**Figure 1.**
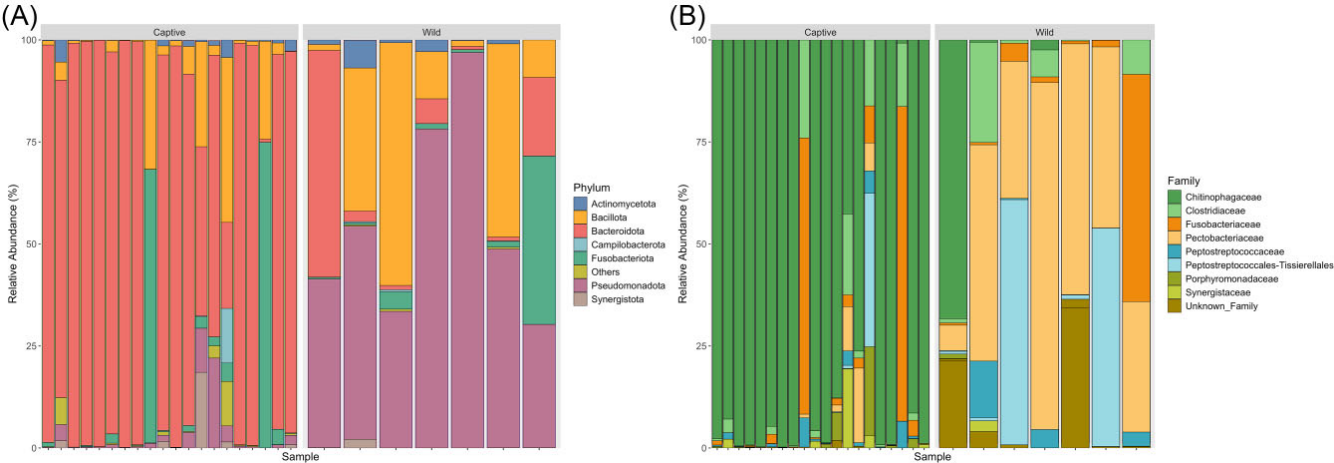
Relative abundance of the most abundant phyla (A), and the top 10 families (B) following 16S rRNA gene sequencing of saltwater crocodile (*C. porosus*) samples from the Northern Territory. Significant differences can be seen in community structure at both a phylum and family level between captive and wild crocodiles.

In total, 28 crocodiles were captured and sampled, 20 animals from a captive population and 8 were sourced from the wild. Sequence data was obtained from 27 animals, and a sum of 452 471 sequences were generated after quality checking and removal of chimeras, giving an average of 16 758 sequences per sample. The taxonomic breakdown of sequence data yielded 16 bacterial phyla, 24 classes, 52 orders, 68 families, 74 genera, 47 species, and 414 ASVs. Differences in proportions of the dominant phyla existed between locations. In captive crocodiles, the most dominate phyla were Bacteroidota (77.8%), Fusobacteriota (8.01%), and Bacillota (7.4%), and for wild crocodiles they were, Pseudomonadota (54.5%), Bacillota (23.6%), Bacteroidota (12.3%), and Fusobacteriota (7.1%) (Fig. 1A). Also, like bacterial phyla, differences existed for the predominate families in the two sets of crocodiles where in captivity Chitinophagaceae (75.9%), Fusobacteriaceae (8.1%), and Clostridiaceae (3.9%) dominated, and in wild animals the most common families were Pectobacteriaceae (40.2%), Peptostreptococcales–Tissierellales (15.3%), Chitinophagaceae (8%), and Fusobacteriaceae (7.1%) (Fig. 1B).

**Figure 2.**
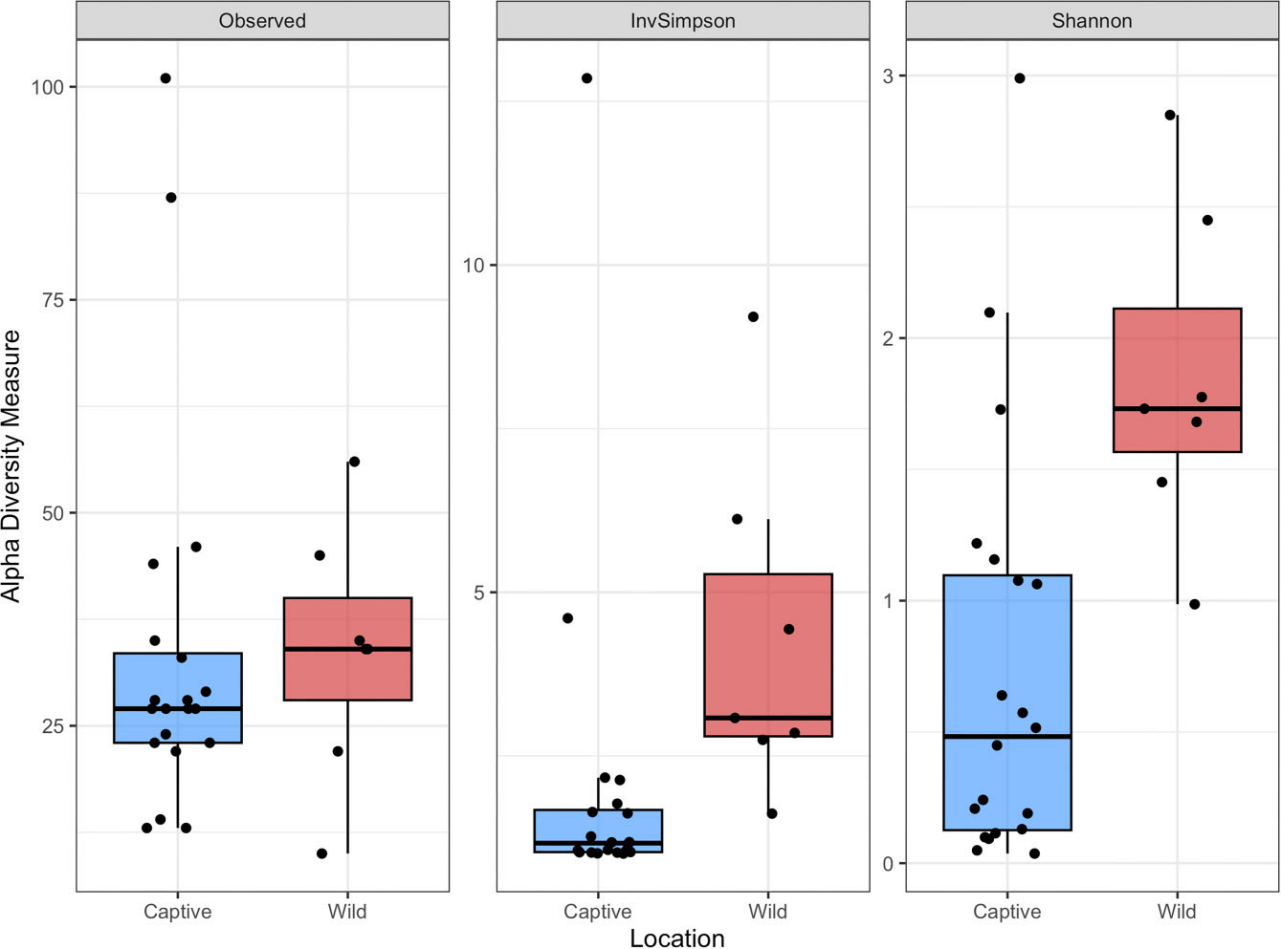
Alpha diversity metrics from saltwater crocodiles (*C. porosus*) from the Northern Territory. *Significant differences* were observed for Inverse Simpson (*P* < .01) and Shannon (*P* = .01) diversity between captive and wild crocodiles.

**Figure 3.**
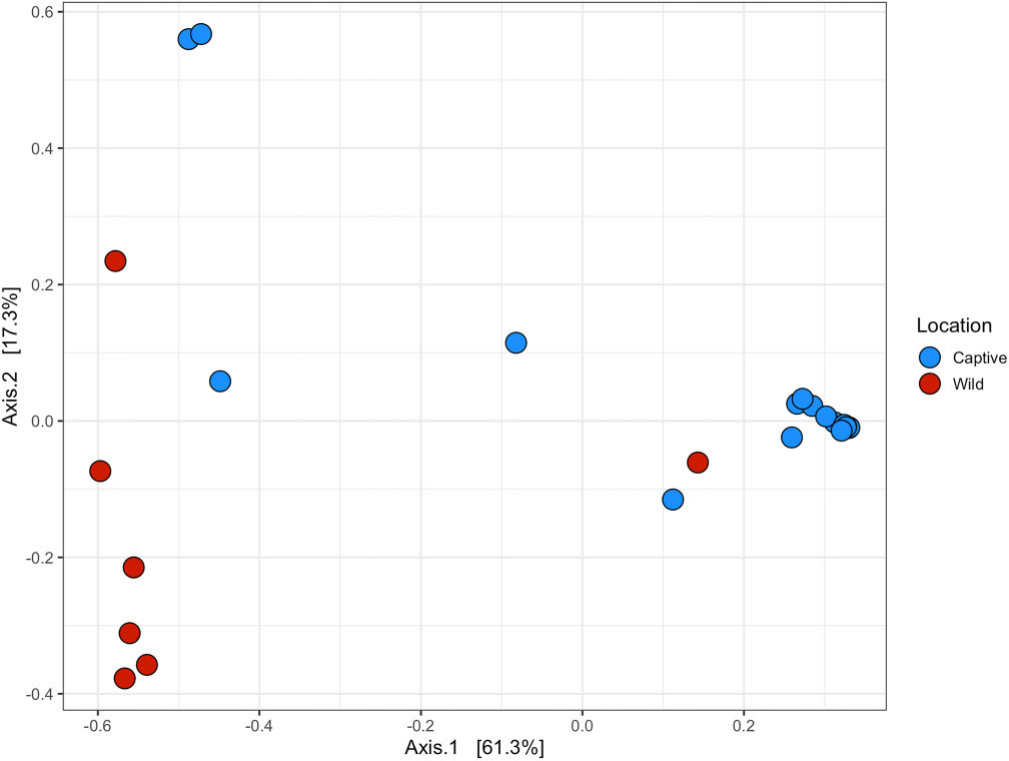
Principal coordinate analysis (PCoA) with Bray–Curtis distances of microbiotas from saltwater crocodiles (*C. porosus*) analysed at the family level from the Northern Territory. PERMANOVA revealed significant differences between captive and wild crocodiles (*P* < .01).

Analysis of alpha diversity revealed that all metrics were nonnormally distributed; Observed (*W* = 0.78, *P* < .01), Inverse Simpson (*W* = 0.63, *P* < .01), and Shannon (*W* = 0.89, *P* = .01), and there were no significant differences between localities for Observed [*W* = 56, *P* < .45, *r* = 0.15 (small)], but there were for Inverse Simpson [*W* = 17, *P* < .01, *r* = 0.56 (large)], and for Shannon diversity [*W* = 19, *P* < .01, *r* = 0.54 (large)] (Fig. 2). For beta diversity, we observed distinct clustering according to the origin of the crocodile and this was supported by significant differences between groups when tested statistically (df = 1, SS_T_ = 2.35, *R*^2^ = 0.35, f.model = 13.71, *P* < .01) (Fig. 3). However, the beta dispersion test yielded a nonsignificant result (df = 1, SS_T_ = 0.22, *R*^2^ = 0.23, f.model = 2.31, *P* = .16) suggesting limited heterogeneity in dispersion. Increased convergence among groups might have been evident had the dataset included more wild specimens, or incorporated additional explanatory factors such as environmental conditions or host genetics.

### Functional omics results

**Figure 4.**
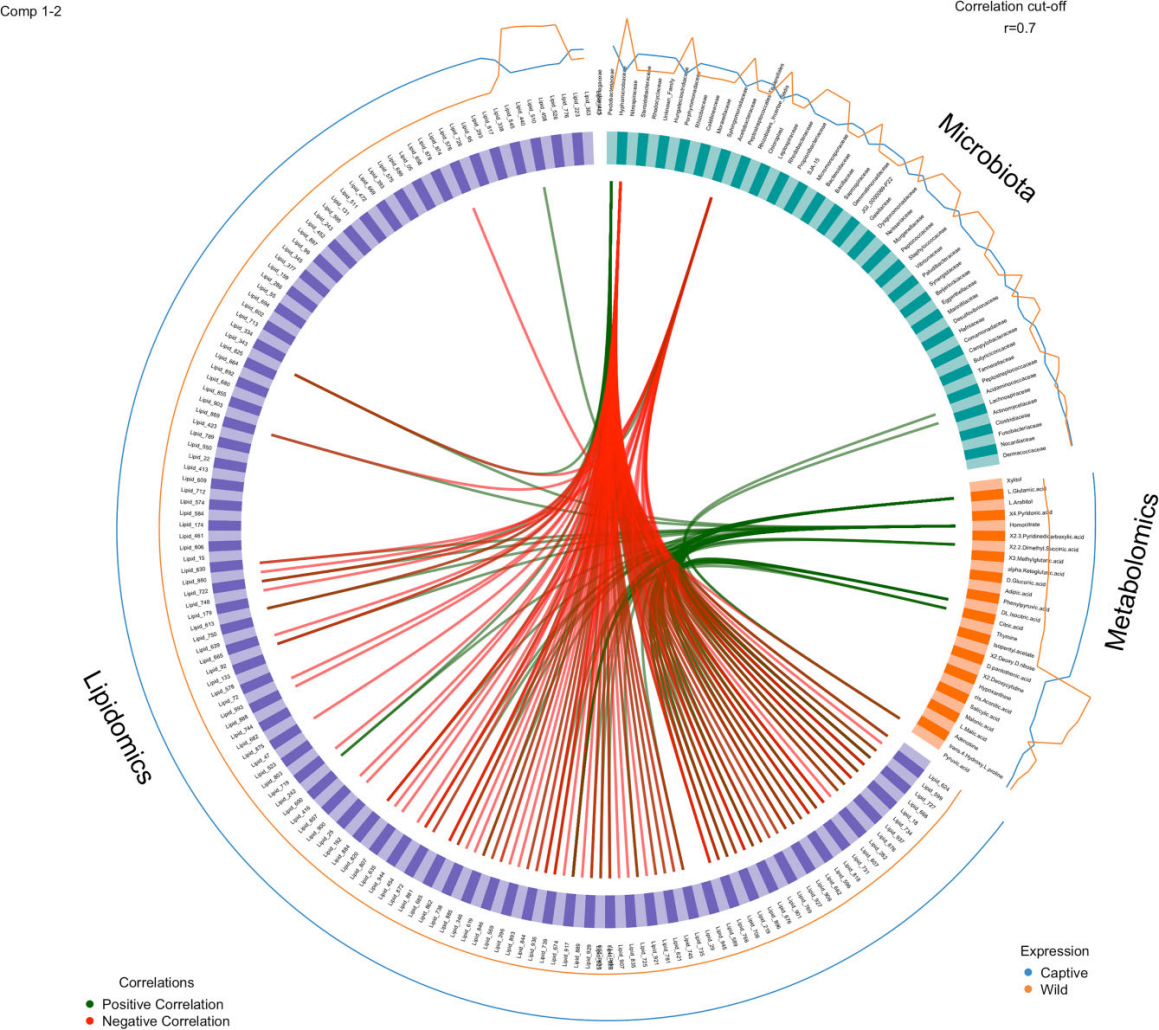
Circos plot from DIABLO performed on the study. The plot represents correlations greater than 0.7 between the variables, which are represented as coloured blocks on the side of the plot; microbiota (teal), metabolomics (orange), and lipidomics (purple). The internal lines show positive (green) and negative (red) correlations while the outer lines show expression levels of each variable in each sample group.

**Figure 5.**
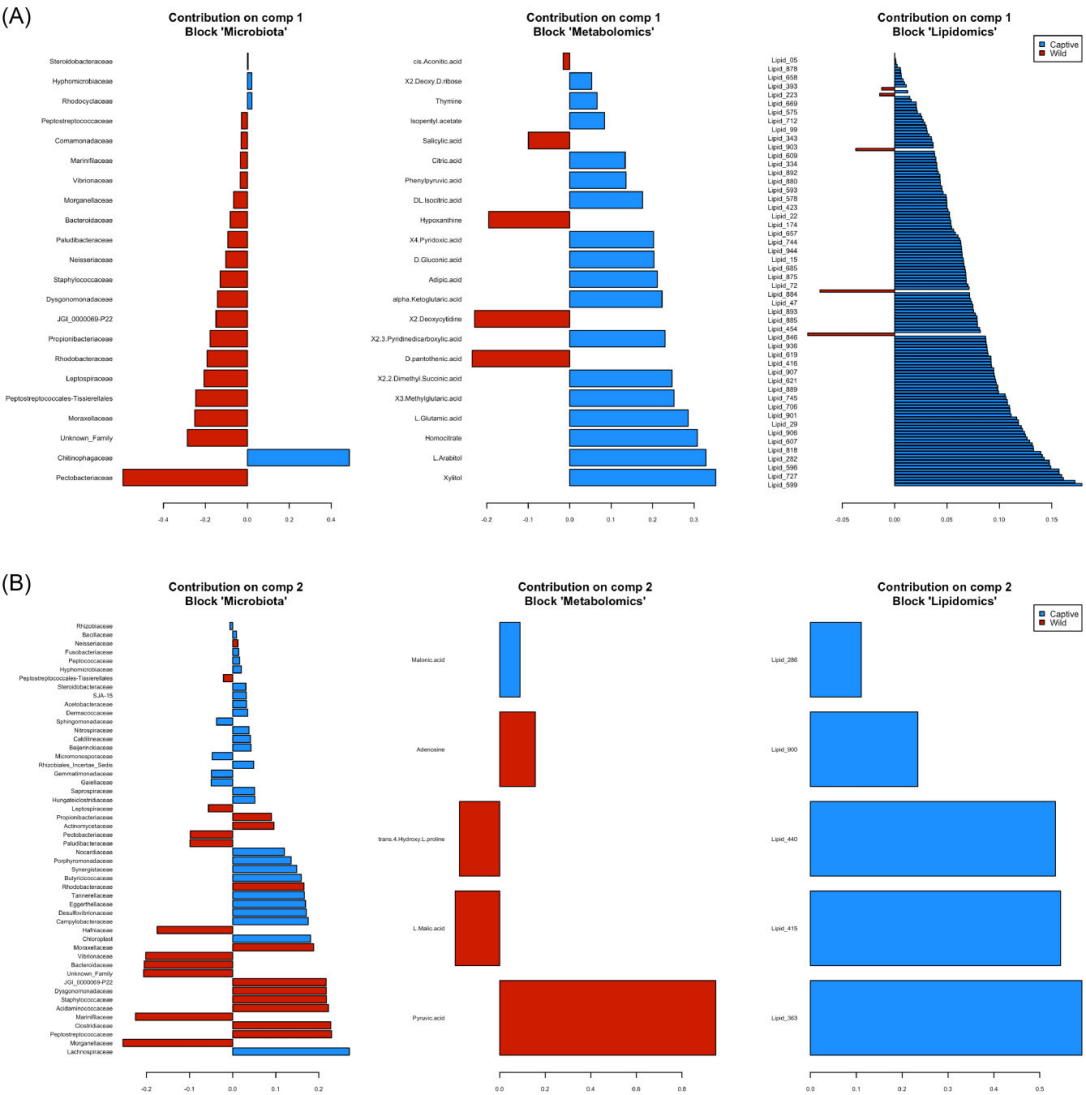
Loading plots for the variables selected by DIABLO performed on the study for component 1 (A) and component 2 (B). The most important variables are ordered from bottom to top.

**Figure 6.**
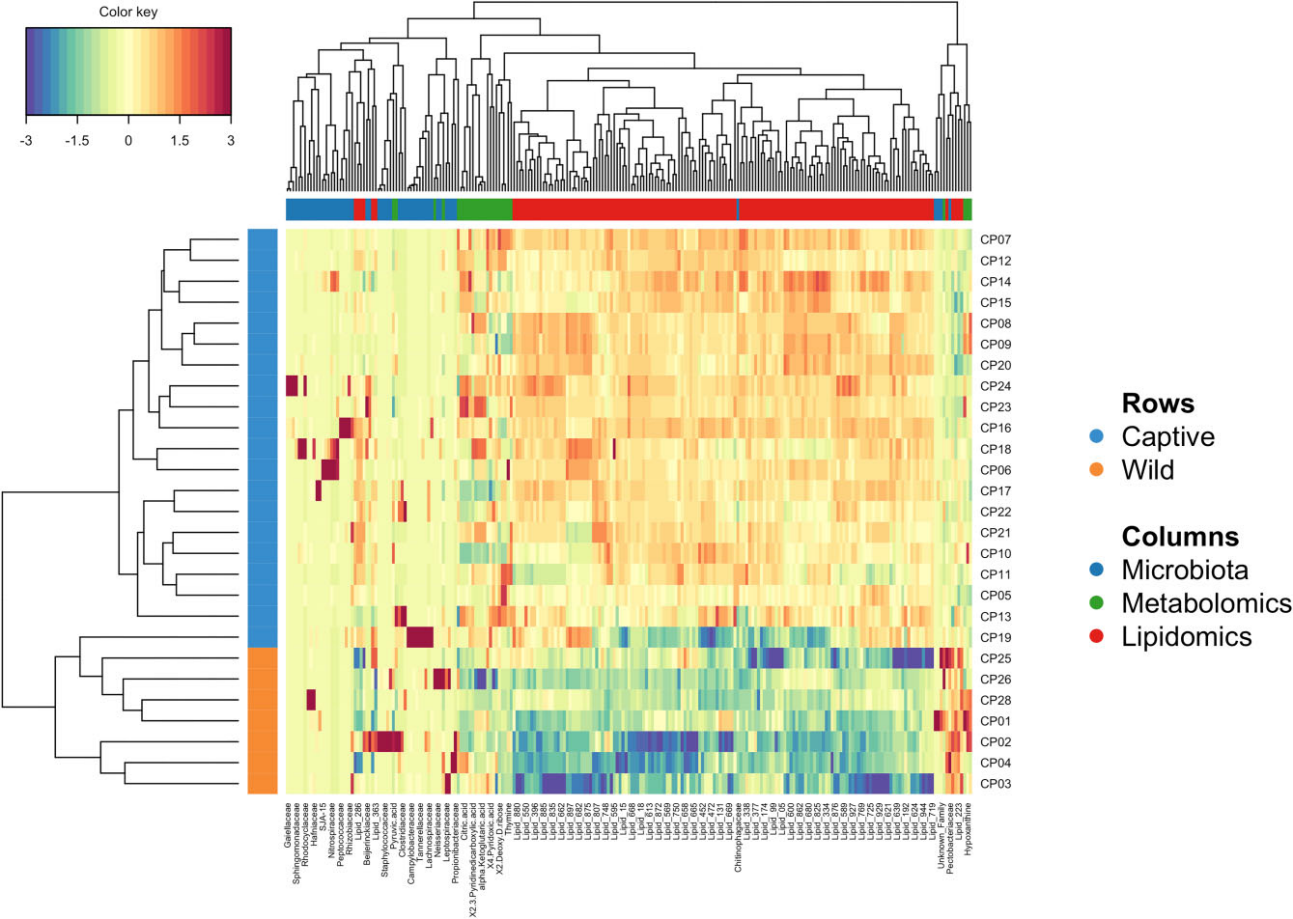
CIM for the variables selected by DIABLO performed on the study. The CIM represents samples in rows and selected features in columns.

The initial dataset that was used for exploration in the model consisted of 53 bacterial Families, 81 metabolomics, and 493 lipidomic compounds. Diagnostic DIABLO plots and correlation circle plots indicated a strong correlation for microbiota composition and lipidomics (0.9) and a moderate correlation for microbiota composition and metabolomics (0.64) (Figs S3 and S4). Furthermore, sample plots (Fig. S5) and arrow plots (Fig. S6) indicated distinct differences existed between crocodile groups for all metrics measured, microbiota, metabolomics, and lipidomics and highlighted the relationship that captivity, and subsequently microbiota composition, has on crocodile physiology. The correlation between the variables from the three different blocks is shown in the Cirosplot, with five bacterial families exerting positive and negative effects on a multitude of metabolomic and lipidomic compounds (Fig. 4). Using loading plots, we were able to identify a signature of bacteria discriminating wild and captive crocodiles (Fig. 5A and B). The families Pectobacteriaceae and Chitinophagaceae were the main bacterial signatures that discriminated captive versus wild crocodiles. Similarly, we identified a range of metabolomic and lipidomic molecules that discriminated between our populations with the majority of these arising from captive animals (Fig. 5A and B). The unique multiomic signature of the crocodiles was depicted using a CIM (Fig. 6) and hierarchical clustering of the samples classified crocodiles into their point of origin, with all individuals correctly classified into their respective groups. To assess the performance of the proposed omics profile we calculated an overall error rate for the majority vote of 0.1 for component 1, which was not improved with the addition of a second component, and an overall error rate for the weighted vote of 0.1 for component 1, which also did not improve with the addition of component 2 to the model. Additionally, the receiver operating characteristic curve analysis showed that the optimal omics profile for microbiota block was 1 (comp 1 *P* < .01; comp 2 *P* < .01), metabolomics was 0.92 (comp 1 *P* < .01; comp 2 *P* < .01), and lipidomics was 1 (comp 1 *P* < .01; comp 2 *P* < .01) (Figs S7 and S8). These results support the above-selected features as representative omics profiles of the crocodiles in this investigation.

**Figure 7.**
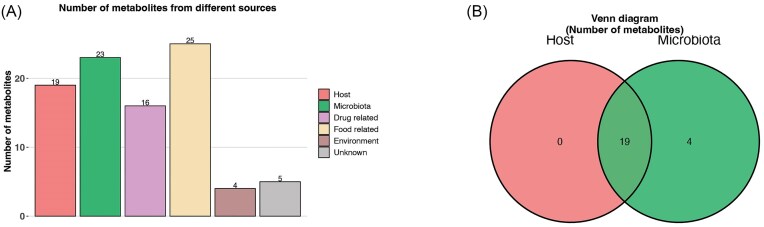
Number of identified metabolites from different sources as determined by MetOrigin performed against the saltwater crocodile (*C. porosus*) genome.

The metabolite host–microbiota origin analysis indicated that of our significant metabolites, 19 may have originated from the host, and 23 may be microbiota-based (Fig. 7A; Table S2). Interestingly, none of the identified metabolites were considered host only, with four of definite microbiota origin (Fig. 7B). There were five metabolites whose origins were unable to be determined (Fig. 7A; Table S2).

## Discussion

The aim of this investigation was to describe the effects that captivity have on the bacteriome, and subsequently the physiology, of saltwater crocodiles. To our knowledge, this is the first study to characterize these traits in a species of crocodile, with the only comparable study to have been conducted in the Chinese alligator (Hu et al. 2022). We were able to demonstrate that captivity alters bacterial community structure in saltwater crocodiles at both a Phylum and Family level. In captive crocodiles, the most dominate phyla were Bacteroidota, Fusobacteriota, and Bacillota, and for wild crocodiles they were, Pseudomonadota, Bacillota, Bacteroidota, and Fusobacteriota. The results of this investigation differ from other analyses involving colonic samples from captive saltwater crocodiles, in which a predominance of Bacillota, Fusobacteriota, and Bacteroidota were described in one study (Willson et al. 2019), and an almost uniform isolation of a single phylum, Pseudomonadota in a second study (Siddiqui et al. 2023). In comparison, faecal/cloacal sampling has shown an overwhelming dominance of Fusobacteriota in farmed American alligators (Keenan et al. 2013), and a mixed population of Fusobacteriota, Bacteroidota, and Bacillota in captive juvenile Siamese crocodiles (Lin et al. 2019). In wild animals, cloacal sampling has revealed a predominance of Pseudomonadota, Fusobacteriota Campylobacterota, and Bacillota in Chinese alligators (Hu et al. 2022), and in American alligators the phyla Fusobacteriota, Bacillota, and Pseudomonadota were the most prevalent (Keenan et al. 2013). Making meaningful comparisons between these investigations and ours is difficult as some studies used deceased animals and sterile dissection to obtain samples directly from the gastrointestinal tract and did not sample cloacal populations. We were not able to use cadavers in our investigation as all captive animals were sourced from a zoological institution and not destined for commercial use. These differences highlight the need to assess microbiota data considering methodology, as it can have a significant effect on interpretation of results (Bartolomaeus et al. 2021).

Like bacterial phyla, differences existed for the predominate families in the two sets of crocodiles, most notably for the family Pectobacteriaceae, which was significantly enriched in wild crocodiles (Figs 1B and 5A). The Pectobacteriaceae are typically pathogens of plants and as such are naturally found in vegetation and soil (Van Gijsegem et al. 2021). An increase in Pectobacteriaceae in wild animals may suggest that free-ranging crocodiles had greater exposure to this family either through accidental ingestion of environmental substrates, or through bioaccumulation by predating on herbivorous prey. Another plausible explanation for the enriched Pectobacteriaceae in wild crocodiles is that specific members of this family are specially adapted to crocodilian guts and form a normal component of the microbiota with important physiological functions. This is evidenced by the negative correlation associated with this family and the lipids octadecadienoic acid (Lipid_624), PC(22 : 1(13Z)/P-18 : 1(11Z)) (Lipid_599), C_50_H_78_N_8_S (Lipid_727), PS(22 : 1(11Z)/0 : 0) (Lipid_668), and LysoPC(18 : 2(9Z,12Z)) (Lipid_18) and their higher concentration in captive crocodiles (Figs 4 and 5A). Of these lipids, only Lipid_18 was able to be definitively identified as food related, with all other compounds either unknown or from host or microbiota origin (Table S2). The physiological consequences and costs or benefits to host fitness associated with a decrease in the relative abundance of the Family Pectobacteriaceae, and the subsequent upregulation of specific lipids as seen in captive crocodiles, remains unknown at this stage. We also saw an increase in the relative abundance of the Family Chitinophagaceae in captive crocodiles (Fig. 1), which is likely attributable to the addition of crab to their diets. The exoskeleton of crabs is comprised predominately of chitin (Chen et al. 2008), and the Chitinophagaceae contains a number of genera that are able to degrade this polymer (Rosenberg 2014). Therefore, increasing the relative proportion of crustaceans in the diet is expected to be a significant selection pressure to preferentially retain microbes with this ability.

The Shannon diversity of saltwater crocodiles differed significantly between wild and captive individuals. These findings are consistent with previous investigations in some vertebrates, which have shown a correlation between decreased gut microbial alpha diversity and captivity (Cheng et al. 2015, Chi et al. 2019, Gibson et al. 2019, Florkowski et al. 2023). Differences in Shannon diversity between our crocodile groups may have been caused by captive husbandry practices that resulted in a microbially depauperate environment. Antibiotic treatments, either administered directly to captive crocodiles, or as residual compounds in farm-raised prey items could significantly diminish gut bacterial diversity (Subirats et al. 2019, Rochegue et al. 2021). Enclosure and other disinfection protocols have been shown to alter gut microbiota composition in a variety of vertebrates and may have played a role in this investigation (Mahnert et al. 2019, Yan et al. 2023, Xu et al. 2024). Other factors that may have contributed to lower Shannon diversity in captive crocodiles include reduced dietary variability (Gillman et al. 2022, Xiao et al. 2022), less frequent contact with a range of environmental substrates (Diaz and Reese 2021), and abnormal social structure leading to increased microbial transfer between individuals (Raulo 2015, Raulo et al. 2021). Interestingly, captivity is not always associated with decreased alpha diversity in animals, with some studies reporting an increase in microbial richness in a variety of species under human care (Alberdi et al. 2021, Malukiewicz et al. 2022, Wang et al. 2021, Xie et al. 2016). This disparity highlights the heterogeneity of microbial community composition in vertebrates and the need to assess individual species to properly assess the temporal dynamics of vertebrate gut microbiotas.

The results of the bacterial analysis of this investigation were consistent with other studies in reptiles, where captivity has been shown to alter gut microbiotas in representatives from all reptilian orders (Fong et al. 2020, Hoffbeck et al. 2024, Tang et al. 2022, Wang et al. 2025, Zhang et al. 2022). However, in some species, such as the Texas horned lizard (*Phrynosoma cornutum*), animals displayed considerable plasticity in their gut microbial composition, such that when captive animals were released back into the wild their intestinal flora quickly assimilated to resemble resident wild animals (Forehand et al. 2025). A similar phenomenon has been observed in Fijian crested iguanas (*Brachylophus vitiensis*) that were headstarted in captivity before release into the wild (Eliades et al. 2021). How captivity alters reptile gut microbiomes is likely due to a range of factors, including species-specific physiological and ecological traits, variation in captive diets and environments, and differing microbial baselines in wild populations. Some reptiles may retain aspects of their natural microbiome due to dietary or habitat similarities in captivity, while others may experience substantial shifts due to altered exposure to environmental microbes, reduced microbial diversity in captive settings, and stress-related physiological changes. Such a multitude of variables make it challenging to generalize the effects of captivity on microbial populations across and even within taxa.

Analysis of serum metabolome showed an enrichment of the alcohol sugars xylitol and l-arabitol in captive crocodiles. Alcohol sugars are commonly derived from plant material such as berries and fruits, or they can be formed by the fermentation of hemicelluloses by microorganisms (Ortiz et al. 2013). These compounds are known to have beneficial health properties in humans including a reduction in constipation, diabetes, and obesity, and by stimulating digestion and the immune system (Awuchi and Echeta 2019, Gasmi Benahmed et al. 2020). However, several negative effects have been attributed to excessive consumption of alcohol sugars, such as irritable bowel syndrome, diarrhoea, nephrolithiasis, urolithiasis, and metabolic acidosis (Awuchi and Echeta 2019, Gasmi Benahmed et al. 2020). Additionally, in human clinical trials xylitol has been shown to reduce the abundance of Bacteroidetes while increasing Bacillota in the gut (Ruiz-Ojeda et al. 2019), which is the opposite to the findings in this study, in which we saw an increased proportion of Bacteroidetes and a decreased proportion of Bacillota in captive crocodiles in comparison to their wild counterparts (Fig. 1). No vegetables or fruits were ever offered to the captive animals and so we cannot be sure why alcohol sugars were considerably higher in this cohort. Furthermore, given that we did not undertake any assessment of gastrointestinal health in these animals we do not know if these compounds are detrimental to crocodilians.

Another significant finding in the assessment of the serum metabolome was the increase in abundance of pantothenic acid in wild crocodiles. Pantothenic acid is an essential dietary factor in vertebrates and deficiencies can lead to a variety of disorders, one of which is dermatitis (Miller and Rucker 2020). Dermatological conditions are common in farmed crocodiles (Lott et al. 2018), and it is plausible that a deficiency in pantothenic acid decreases the hosts barrier defences and makes them more susceptible to secondary bacterial or fungal infections. Further research is required to determine if supplementing captive crocodiles with pantothenic acid can reduce the incidence of dermatitis.

A limitation of this investigation was the use of cloacal swabs to sample the gut microbiota, which may affect the resolution and completeness of our findings. Bacterial communities vary along different regions of the gastrointestinal tract, and cloacal swabs may not fully represent the diversity or composition found deeper within the gut (Backhed et al. 2005). While swabs are minimally invasive and practical for fieldwork, they may capture only a subset of bacterial taxa, potentially underrepresenting less abundant or region-specific organisms (Fair et al. 2019). Furthermore, this sampling method lacks the temporal depth needed to assess bacterial dynamics over time, limiting insights into causality or bacteriome stability (Vandeputte et al. 2021). Despite these constraints, cloacal swabbing remains the most feasible and ethically acceptable method for live sampling of wild and captive protected species such as crocodiles in Australia. Consequently, the results should be interpreted with these methodological limitations in mind.

Sample sizes in this study were uneven across groups due to the considerable challenges involved in capturing wild saltwater crocodiles. Trapping success was highly variable and constrained by environmental conditions, animal behaviour, and the logistical and safety risks inherent in working with large, potentially dangerous reptiles. The capture rates in this investigation reflect the difficulty of obtaining balanced sample sizes in field-based microbiome research on apex predators. Despite these limitations, the data collected are consistent with other published work and provide valuable insights into crocodilian microbiota. These constraints are acknowledged and have been considered in the analysis and interpretation of the findings. Future research should aim to extend fieldwork across multiple seasons and locations to increase sample sizes, improve group balance, and capture temporal variation in microbial communities.

In this investigation, we were able to demonstrate that captivity results in alteration of the saltwater crocodile cloacal bacteriome as well as changes in the metabolome. We were also able to exhibit a clear link between the bacteriome and physiology in crocodilians. However, we were not able to establish what effects, if any, these differences have on host fitness and adaptability. While it was clear that significant differences existed between wild and captive animals, it is possible that these differences are inconsequential when it comes to overall physiological performance of the host. To better elucidate the magnitude of these findings longitudinal investigations involving captive, wild, and captive-bred for release animals are required. Longitudinal studies on animal microbiotas are crucial for understanding population dynamics and how microbial communities change over time in response to season, diet, and environmental fluctuations. They can also aid in monitoring health status and may act as sentinels for early detection of disease or stress in populations. Established microbiota investigations may also offer ecological insights by providing comprehension into how microbes interact with hosts and their environments, which can better inform conservation strategies and ecosystem management. Finally, future investigations should focus on determining the plasticity of crocodilian microbiotas, and whether if captive animals are released into the wild their gut microbial populations will assimilate to resemble free-ranging conspecifics.

## Supplementary Material

fiaf114_Supplemental_Files

## Data Availability

All data presented here and in the supplementary material have been submitted to The National Center for Biotechnology Information (www.ncbi.nlm.nih.gov) PRJNA1253344 and can be accessed on reasonable request from the authors.
