# Supplementary material for: Unravelling the impacts of captivity on saltwater crocodile (Crocodylus porosus) cloacal bacterial communities and physiology
Source: FEMS Microbiol Ecol. 2025 Nov 19;101(12):fiaf114. doi: 10.1093/femsec/fiaf114 (PMC12658888; doi:10.1093/femsec/fiaf114)
Supplement: fiaf114_Supplemental_Files [file fiaf114_supplemental_files.zip › Supplementary Material.docx]

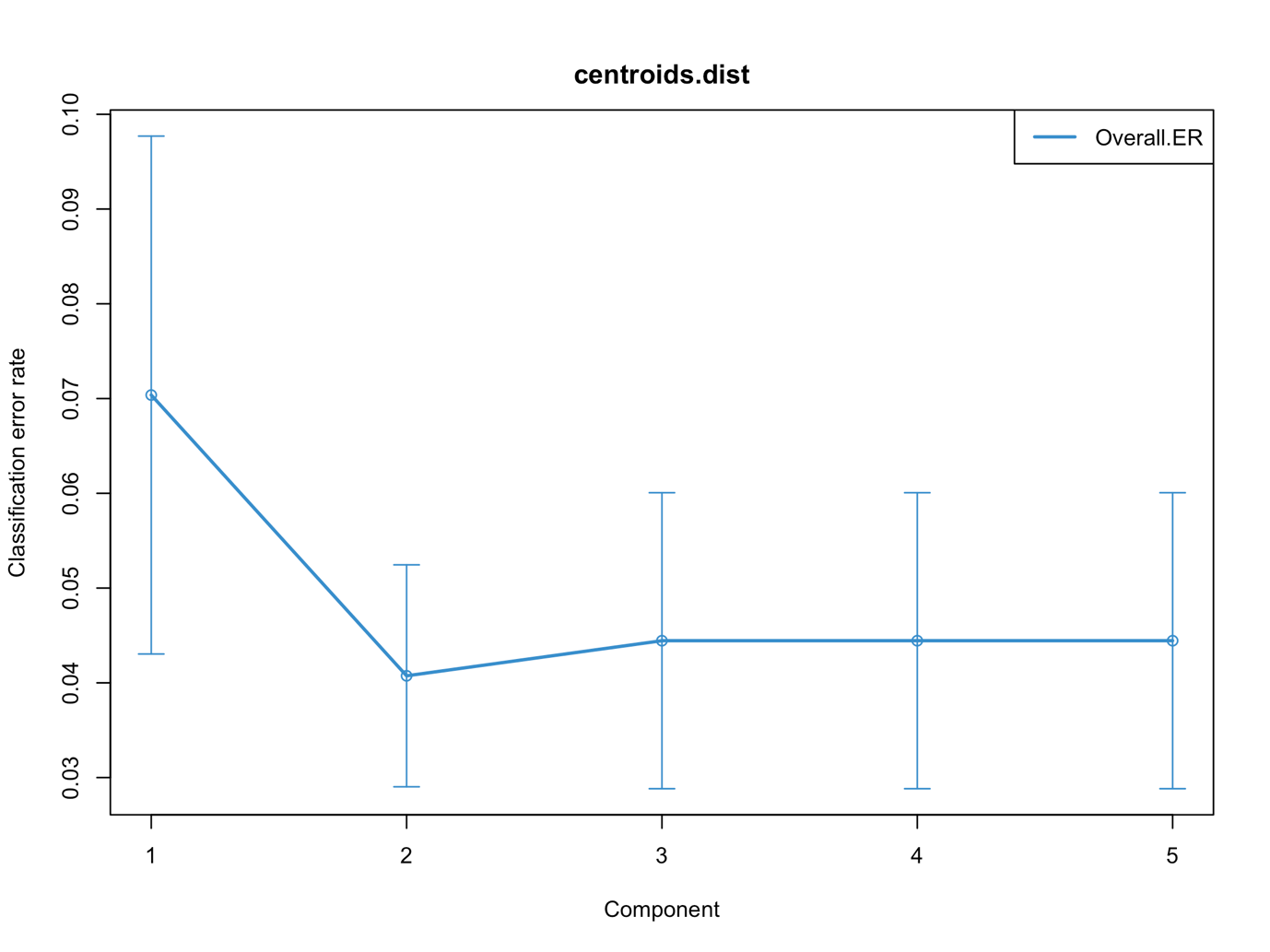


**Figure S1.** Plot exploring evaluation of the fitted SPLSDA model. Based on the appearance of this plot 2 components were chosen for analysis.


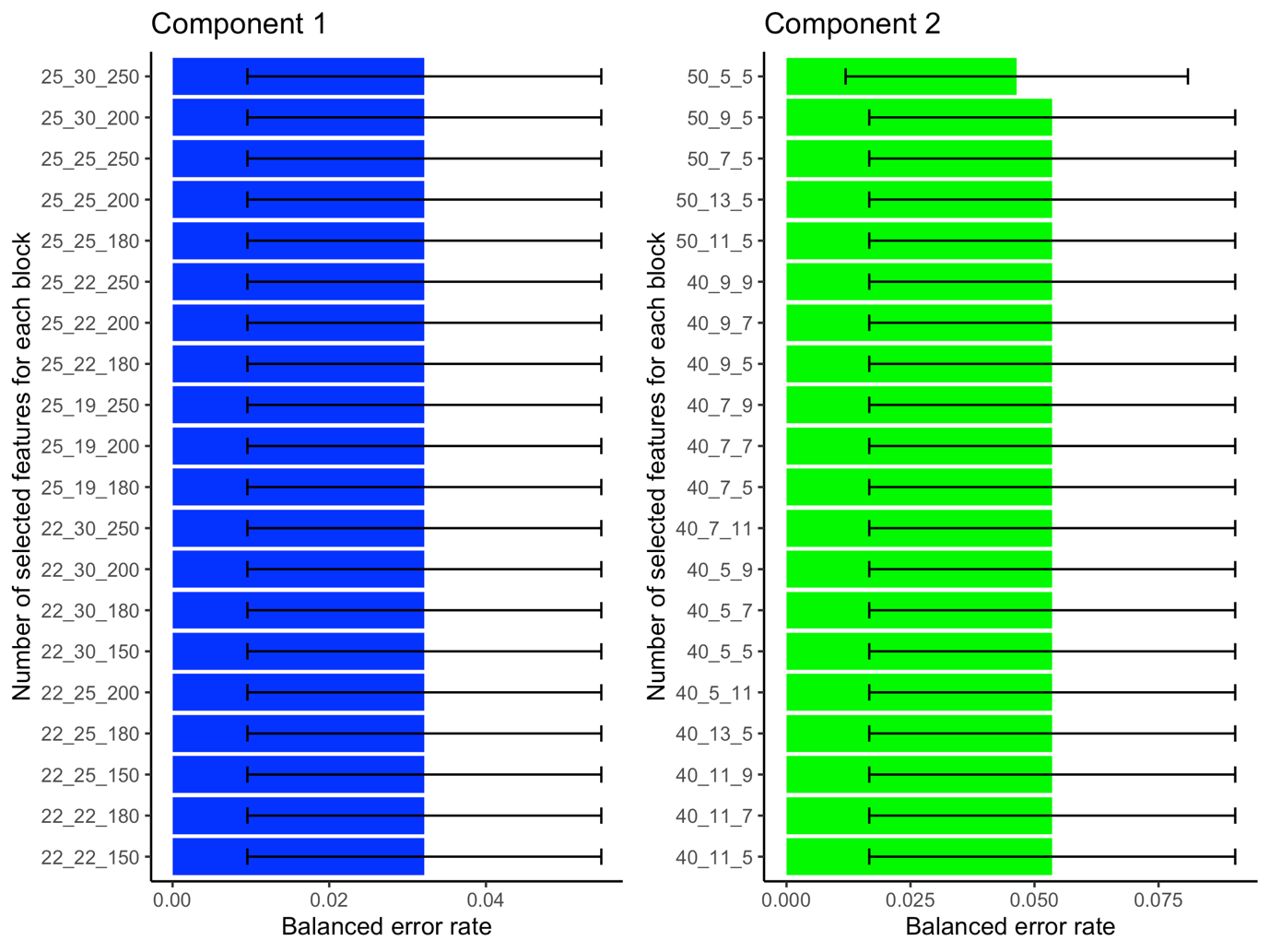


**Figure S2.** Feature selection stability assessment indicating that error rate did not change with the addition or removal of features.

**
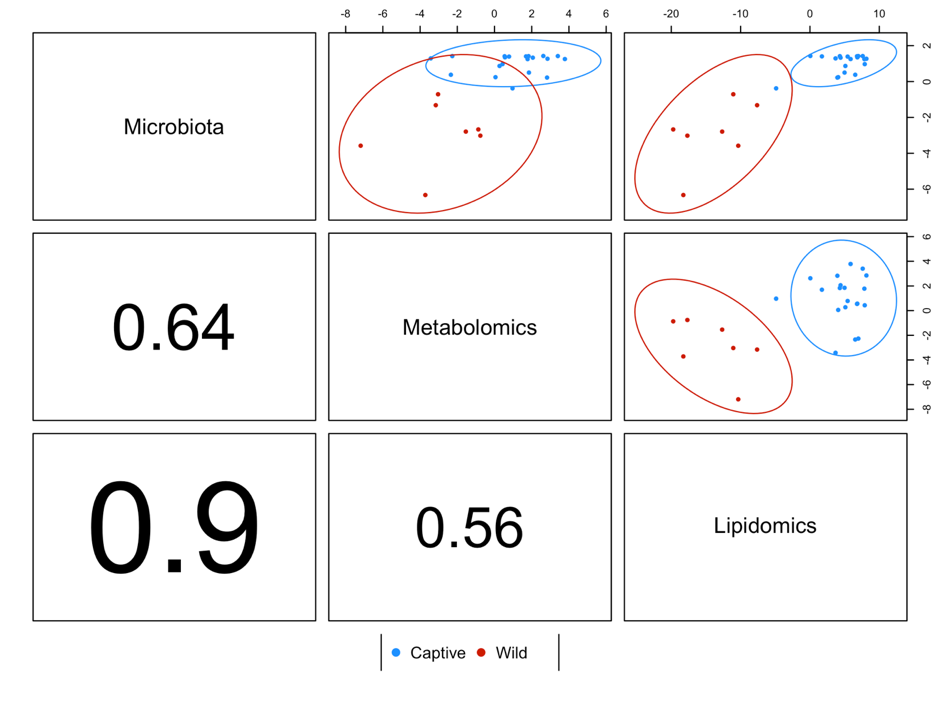
**

**Figure S3.** Diagnostic plot from multiblock SPLSDA applied to the data. Samples are represented by component 1 for each data set (Microbiota, Metabolomics and Lipidomics). Samples are coloured by location (Captive and Wild). The bottom left numbers indicate the correlation coefficients between each dataset.

**
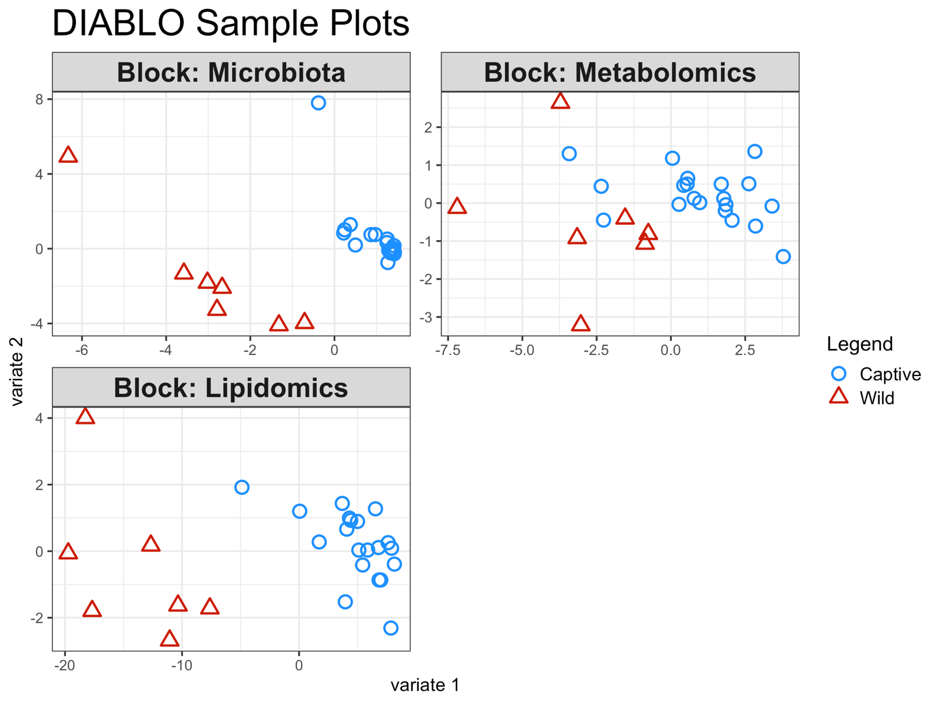
**

**Figure S4.** Sample plot from multiblock sPLSDA performed on the data. The samples are plotted according to their scores on the first 2 components. Samples are coloured by location (captive and wild) and it shows the degree of agreement between the different datasets and the discriminative ability of each.


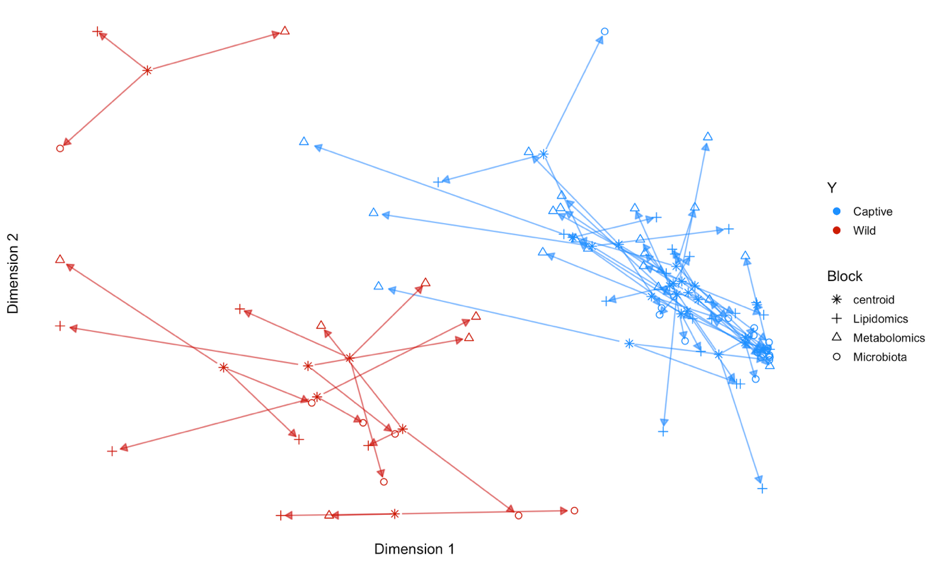


**Figure S5.** Arrow plot from multiblock sPLSDA performed on the data. Arrows further from their centroid indicate some disagreement between the datasets. Samples are coloured by location (Captive and Wild).


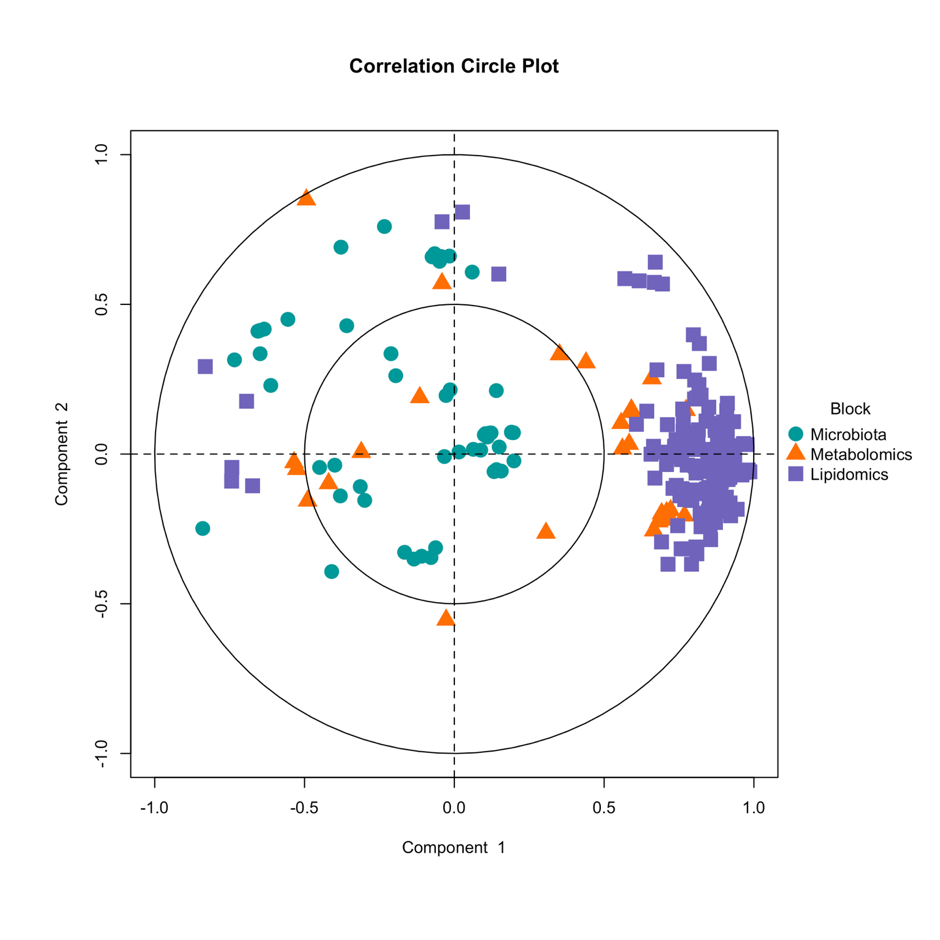


**Figure S6.** Correlation circle plot from multiblock sPLSDA performed on the data. Clusters of points indicate a strong correlation between variables.


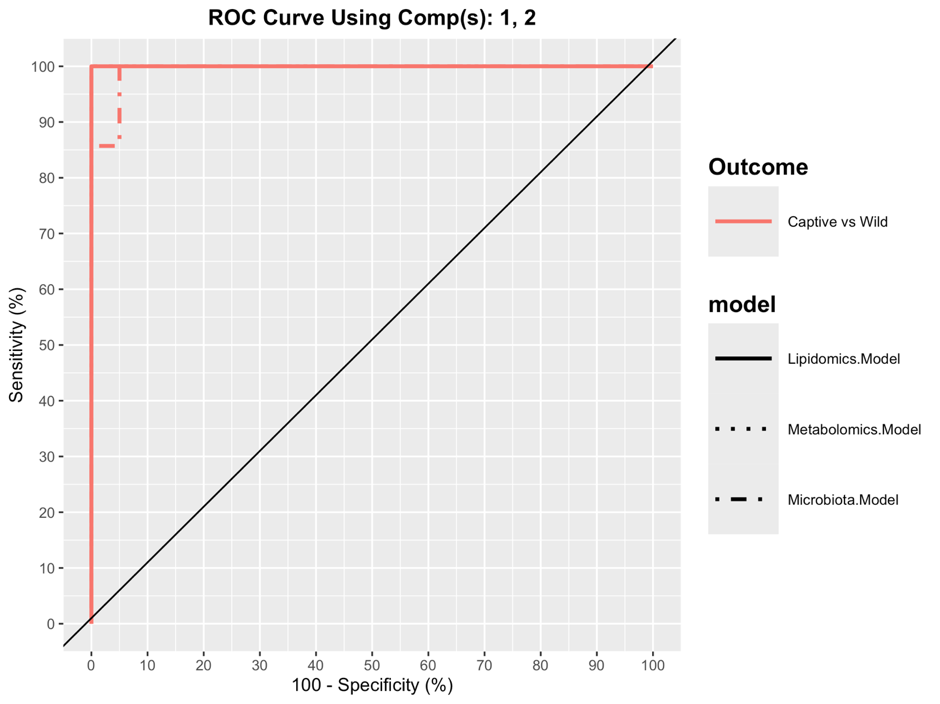


**Figure S7.** Receiver operating characteristic (ROC) and area under the curve (AUC) based on multiblock sPLSDA performed on the data for microbiota, metabolomics, and lipidomics.


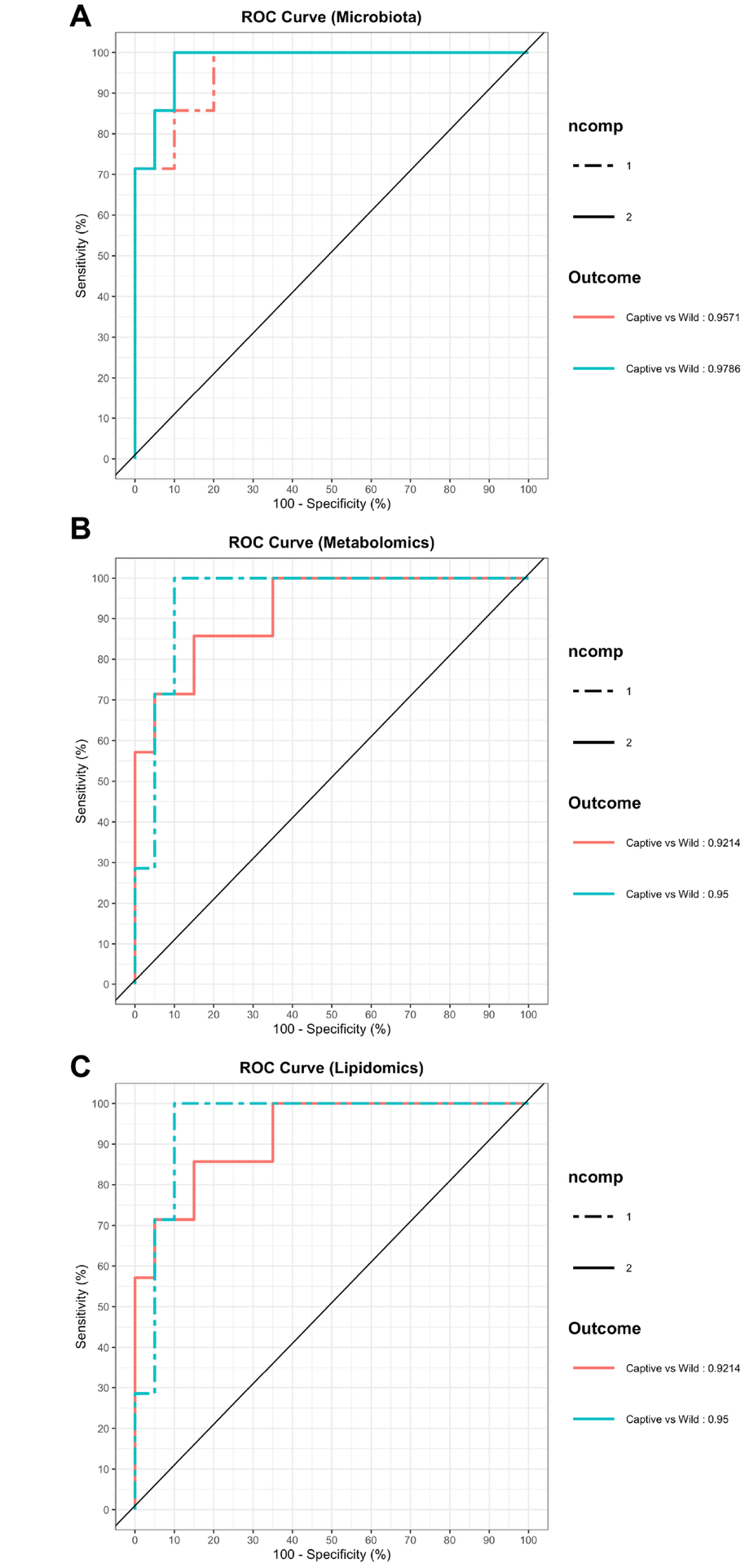


**Figure S8.** Sensitivity analysis for our parameters microbiota **(A)**, metabolomics **(B)**, and lipidomics **(C)** indicating that for all analytes sensitivity increased with the addition of two components.
